# Testing paradox may explain increased observed prevalence of bacterial STIs among MSM on HIV PrEP: A modeling study

**DOI:** 10.1073/pnas.2524944122

**Published:** 2025-10-29

**Authors:** Laura Müller, Piklu Mallick, Antonio B. Marín-Carballo, Philipp Dönges, Robyn J. N. Kettlitz, Carolina J. Klett-Tammen, Mirjam Kretzschmar, Viola Priesemann, Seba Contreras

**Affiliations:** ^a^Max Planck Institute for Dynamics and Self-Organization, Göttingen 37077, Germany; ^b^Institute for the Dynamics of Complex Systems, University of Göttingen, Göttingen 37077, Germany; ^c^University of Granada, Granada 18071, Spain; ^d^Department of Epidemiology, Helmholtz Centre for Infection Research, Brunswick, Lower Saxony 38124, Germany; ^e^PhD Program “Epidemiology” Brunswick-Hanover, Lower Saxony 38124, Germany; ^f^University Medical Center Utrecht, Utrecht University, Utrecht 3584, The Netherlands; ^g^Center for Complex Systems Studies, Utrecht University, Utrecht 3584, The Netherlands; ^h^Interdisciplinary Center for the Mathematical Modeling of Infectious Disease Dynamics, University of Münster, Münster 48149, Germany

**Keywords:** HIV PrEP, MSM, STI, asymptomatic screening, public health

## Abstract

HIV pre-exposure prophylaxis (PrEP) is transforming global HIV prevention, but its implementation coincides with observations of rising bacterial sexually transmitted infection (STI) rates among men who have sex with men, raising questions about whether PrEP is preventing one epidemic while facilitating others. To reconcile this apparent contradiction, we developed a minimal dynamical model of the simultaneous transmission of HIV and *chlamydia* (as an example of a curable STI). The model integrates three key mechanisms: 1) risk-mediated self-protective behavior, 2) reduction in condom use among PrEP users, and 3) PrEP-related asymptomatic STI screening. We show that these mechanisms can generate a “testing paradox:” True STI prevalence may decline while observed trends rise. This paradox emerges because increased PrEP uptake amplifies screening intensity, which can lower transmission but simultaneously inflate detection. By systematically mapping the parameter space of PrEP uptake, screening frequency, and risk perception, we identify broad and plausible conditions under which the paradox arises. Our findings reconcile conflicting epidemiological evidence and remark that the net effect of PrEP on STI dynamics depends critically on asymptomatic screening strategies. These results highlight the potential dual role of PrEP programs in reducing both HIV and bacterial STI incidence, while emphasizing the need to align screening and treatment policies to maximize benefits and minimize risks, e.g., antimicrobial resistance.

## Significance

The widespread adoption of HIV pre-exposure prophylaxis (PrEP) among men who have sex with men has coincided with rising rates of other sexually transmitted infections (STIs), raising concerns about unintended public health consequences. Our study suggests that observed increases in STI cases may be attributed to the intensified asymptomatic screening rather than genuine increases in transmission. This “testing paradox” reconciles conflicting epidemiological evidence and highlights the importance of distinguishing between observed and actual infection trends when evaluating PrEP’s public health impact.

HIV pre-exposure prophylaxis (PrEP) has been transformative in reducing HIV infection risk for users (1, 2). Given its high efficacy and effectiveness at preventing HIV infection, high PrEP coverage is associated with substantial decreases in HIV incidence among risk groups, especially men who have sex with men (MSM) (3, 4). However, behavioral changes driven by risk compensation among MSM on PrEP make them more likely to engage in risky sexual behaviors, heightening their vulnerability to acquiring other sexually transmitted infections (STIs) that are perceived as less severe, e.g., *Chlamydia trachomatis* (CT) and *Neisseria gonorrhoeae* (NG) (5, 6). Although the STIs above are curable [in principle, see e.g., antibiotic-resistant strains of gonorrhea (7)], higher prevalence of such common STIs may drastically change the HIV infection risk landscape by increasing susceptibility at the population level due to facilitation effects in coinfections (8–10). Therefore, maintaining a low incidence of such STIs should remain a priority, especially in populations with low PrEP adherence (11, 12).


[1] $$\begin{array}{cccc} \frac{\mathrm{dS}}{\mathrm{dt}} & =-\underset{\text{contagion}}{\underbrace{\Lambda S}}+\underset{\text{immunity loss}}{\underbrace{\gamma I^{a}+\tilde{\gamma }T}}+\underset{\text{recruitment rate}}{\underbrace{\Phi }}-\underset{\text{deaths}}{\underbrace{\mu S}}-\underset{\text{total influx}}{\underbrace{\Sigma }} & :\; & \text{susceptible,} \end{array}$$

[2] $$\begin{array}{cccc} \frac{dI^{a}}{\mathrm{dt}} & =\underset{\text{asymp. contagion}}{\underbrace{\psi \cdot \Lambda S}}-\underset{\text{recovery}}{\underbrace{\gamma I^{a}}}-\underset{\text{testing and treatment}}{\underbrace{\lambda_{a}\left(P,H\right)I^{a}}}-\underset{\text{deaths}}{\underbrace{\mu I^{a}}}+\underset{\text{asymp. influx}}{\underbrace{\psi \Sigma }} & :\; & \text{asymptomatic infection,} \end{array}$$

[3] $$\begin{array}{cccc} \frac{dI^{s}}{\mathrm{dt}} & =\underset{\text{symptomatic contagion}}{\underbrace{(1-\psi )\cdot \Lambda S}}-\underset{\text{testing and treatment}}{\underbrace{\lambda_{s}\left(P,H\right)I^{s}}}-\underset{\text{deaths}}{\underbrace{\mu I^{s}}}+\underset{\text{symptomatic influx}}{\underbrace{(1-\psi )\Sigma }} & :\; & \text{symptomatic infection,} \end{array}$$

[4] $$\begin{array}{cccc} \frac{\mathrm{dT}}{\mathrm{dt}} & =\underset{\text{testing and treatment}}{\underbrace{\lambda_{a}\left(P,H\right)I^{a}}}+\underset{\text{testing and treatment}}{\underbrace{\lambda_{s}\left(P,H\right)I^{s}}}-\underset{\text{recovery}}{\underbrace{\tilde{\gamma }T}}-\underset{\text{deaths}}{\underbrace{\mu T}} & :\; & \text{treated/recovered,} \end{array}$$

[5] $$\begin{array}{cccc} \Lambda & =\beta_{0}^{\text{STI}}\left((1-m(H))(1-P)+(1-\xi \cdot m(H))P\right)\left(I^{a}+I^{s}\right) & :\; & \text{force of infection,} \end{array}$$

[6] $$\begin{array}{cccc} \lambda_{a}\left(P,H\right) & =\underset{\text{risk-related testing}\;\text{| self-reporting}}{\underbrace{\lambda_{H}\left(H\right)\cdot \left(1-P\right)}}+\underset{\text{PrEP-related testing rate}}{\underbrace{\lambda_{P}\cdot P}} & :\; & \text{asymptomatic testing rate,} \end{array}$$

[7] $$\begin{array}{cccc} \lambda_{s}\left(P,H\right) & =\underset{\text{self-reporting rate}}{\underbrace{\lambda_{0}}}+\underset{\text{testing rate for asymptomatic}}{\underbrace{\lambda_{a}\left(P,H\right),}} & :\; & \text{symptomatic testing rate,} \end{array}$$

[8] $$\begin{array}{cccc} \lambda_{H}\left(H\right) & =k_{\text{or}}\cdot \beta_{0}^{\text{HIV}}\left(1-m\left(H\right)\right)H & :\; & \text{risk-related testing rate,} \end{array}$$

[9] $$\begin{array}{cccc} m(H) & =m_{\text{min}}+\left(m_{\text{max}}-m_{\text{min}}\right)\left(1-\exp\left(\frac{-H}{H_{\text{max}}}\right)\right) & :\; & \text{mitigation function.} \end{array}$$

Intuitively, risk compensation and the shared transmission route between HIV and the STIs above support the association between PrEP initiation and increased STI rates (13–17). However, findings vary: While several studies report increases in STI rates after PrEP initiation (15, 18–23), others observe no change or infection-specific patterns (24–26). Besides potential selection bias in PrEP cohorts and already ongoing rise in STI trends (27, 28), a noncausal explanation for the increased STI rates observed in some settings may be that a larger fraction of the STIs are detected due to more frequent screening in PrEP programs (16, 28–31). Asymptomatic screening for common STIs is part of the PrEP official recommendations (12, 32, 33), allowing infectious individuals to be detected, receive antibiotic treatment, and recover more quickly. Through this mechanism, around 40% of new CT and NG infections can be averted in a baseline scenario with moderate risk compensation and biannual STI screening—which can be reduced an additional 50% if screening each quarter instead (29). However, increased screening can permanently increase the number of infections detected while decreasing the true prevalence of the disease. This duality between true and observed trends (i.e., positive tests) challenges the interpretation of epidemiological data and thereby the assessment of PrEP as a public health intervention (34, 35).

Mathematical modeling of transmission dynamics is a powerful tool to assess the impact of interventions on variables that cannot be fully observable. Consequently, they have proven to be of great use in the study of STIs and in the assessment of the cost-effectiveness of PrEP programs (3, 36–39). While the contribution of PrEP programs to HIV control and elimination has been modeled in great detail, the interplay of STI prevalence and PrEP uptake has mostly been studied empirically, e.g., refs. 15, 17, 27, 40, and 41. One noteworthy exception is the work of Jenness et al. (29), where a network-based model is used to analyze how PrEP could influence STI prevalence via a combination of behavioral risk compensation and PrEP-related STI screening. We go one step further here and pose the following question: Under what conditions can PrEP-related screening and risk perception lead to paradoxical dynamics, where the actual prevalence of STIs declines while the number of positive tests increases?

In this study, we introduce a compartmental model to represent the simultaneous spread of HIV and CT (as an example of a curable STI) in a population of MSM. Our model integrates three mechanisms: 1) risk-mediated self-protective behavior among MSM who are not on PrEP, 2) condom use reduction due to risk compensation post-PrEP initiation, and 3) PrEP-related asymptomatic STI screening at variable frequencies. Our aim is to determine when the interplay between risk perception, PrEP adoption, and screening rate leads to the paradox described above in high-income settings, i.e., where PrEP adoption is high and there are no barriers to its acquisition. We first show that, similar to Jenness et al. (29), higher PrEP coverage may help reduce the incidence of CT if asymptomatic screening is frequent enough, and explore how this effect varies when the risk awareness among MSM not on PrEP varies. We then show that the paradox is indeed present and widespread in various scenarios when comparing real and observed prevalence. The paradox is not present when analyzing changes in the true positivity rate, with one caveat: One must know the total number of tests performed. Our findings offer insights into potential paradoxical dynamics and may thereby help navigate the complexities of interpreting surveillance data.

## Methods

We propose a deterministic compartmental model to represent the simultaneous spread of HIV and CT in a high-infection-risk group of MSM, including the effects of PrEP, HIV infection risk perception, and asymptomatic screening for STIs. The dynamics in this multipathogen system are governed by two key variables: the overall PrEP uptake P and the HIV-infection-risk awareness H among the high-infection-risk population. In our framework, individuals on PrEP fully comply with the screening requirements and administration recommendations. High infection risk is defined as a high number of new sexual partners and overall low use of condoms, which translates to high base spreading rates for STIs.

We hypothesize that individuals who are not on PrEP adapt their behavior according to their perceived HIV infection risk (42, 43). This adaptation implies that these individuals would adopt mitigation measures $m\left(H\right)$ to reduce the risk of infection upon contact and seek asymptomatic STI screening at a rate $\lambda_{H}\left(H\right)$, both monotonically increasing with H. PrEP users, on the other hand, are required by public health plans to test for HIV and other STIs at a rate $\lambda_{P}$, which varies from country to country (32, 44). They also behave differently: They adopt reduced mitigation measures, denoted by ξm(H), as a result of risk compensation. In this way, PrEP uptake feeds back into both the effective transmission rate of curable STIs and the total STI testing rate. Since our primary focus is on the highest-risk group, we assume that individuals do not further assimilate risk and therefore set the risk assimilation parameter ξ=0 (see Table 2). Alternative values of ξ are explored in *SI Appendix*, Text 8.

Our model incorporates the possibility that CT can manifest either as notably symptomatic or asymptomatic. We assume that asymptomatic individuals are detected through testing only if they undergo screening as a response to their risk awareness ($\lambda_{H}$) or as part of routine PrEP-related screening ($\lambda_{P}$). On the other hand, symptomatic individuals additionally seek testing due to symptom onset at a rate $\lambda_{0}$, which is defined as the inverse of the median incubation period ($1/l_{\mathrm{STI}}$) (see Table 1). In either case, once detected, positive individuals receive treatment (antibiotics and counseling) so that they are effectively removed from the pool of infections and recover. We also assume that there is a short period of immunity after treatment, either due to immune response or behavioral changes.

**Table 1.** Model variables

Variable	Definition	Value	Units
S	Fraction of the population susceptible to curable STIs		
$I^{a}$	Fraction of the asymptomatic population infectious with curable STIs		
$I^{s}$	Fraction of the symptomatic population infectious with curable STIs		
T	Fraction of the population treated against curable STIs		
H	Fraction of the population that is risk aware		
P	Fraction of the population that is on HIV PrEP		
N	STI prevalence (fraction of the population currently infectious)	Eq. **10**	
$N^{\text{obs}}$	Observed STI cases (daily positive tests)	Eq. **11**	
$\lambda_{0}$	Self-reporting rate for symptomatic STI	$1/l_{\text{STI}}$	day-1
$\lambda_{a}\left(P,H\right)$	Testing rate (asymptomatic) for STI	Eq. **6**	day-1
$\lambda_{s}\left(P,H\right)$	Testing rate (symptomatic) for STI	Eq. **7**	day-1
$\lambda_{H}\left(H\right)$	Risk-related testing rate for STI or self-reporting	Eq. **8**	day-1
m	Mitigation, self-regulation of contagious contacts	Eq. **9**	-

To incorporate demographic dynamics into the model, we introduce rates Φ and μ, that represent recruitment and withdrawal from the sexually active population. The recruitment term represents the entry of new individuals into the high-infection-risk MSM population and serves a similar role to a birth rate in traditional models. The condition μ=Φ yields a demographic equilibrium stabilizing the population size. Last, although we employ parameters based on CT, our analysis seeks to shed light on the transmission of any of the four curable STIs, which, for modeling purposes, differ only in parameter values. Hereafter, subindexes and references to STI in our model refer to CT.

### Model Equations.

**Fig. 1.**
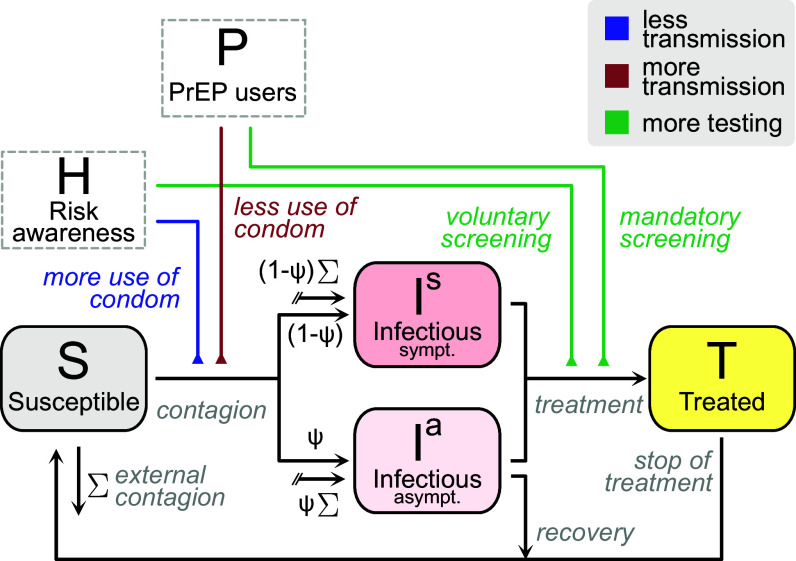
Minimal susceptible-infectious-treated-susceptible model for CT transmission among high-risk MSM, influenced by HIV risk perception and PrEP uptake. This model distinguishes between symptomatic and asymptomatic infections and incorporates three feedback mechanisms: i) increased risk awareness leads to higher condom use (blue, reducing transmission rates), ii) lower condom use among PrEP users (red, increasing transmission rates), and iii) enhanced asymptomatic screening due to both risk perception and PrEP uptake (green, increasing testing rates). Our model also incorporates an additive influx of externally acquired infections Σ, distributed between the $I^{a}$ and $I^{s}$ proportional to the share of cases that are naturally asymptomatic ψ (or its complement). A risk-stratified variation of this model is studied in *SI Appendix*.

Given that the spreading dynamics of HIV and CT have different characteristic times (e.g., as per their generation intervals and spreading rates), we resort to the method of timescale separation to simplify our analysis: When the focus is on the dynamics of CT, HIV incidence and risk awareness do not vary substantially. As a result, we treat the fraction of the population that is on PrEP P and HIV prevalence, thus the population’s infection risk awareness H, as constant inputs when simulating the STI dynamics. A flow diagram of the model is shown in Fig. 1.

For the main text of this article, we focus our analysis on a single high-infection-risk group of MSM in high-income settings, where the effective PrEP uptake is limited only by willingness and compliance. This enables the derivation of analytical solutions for the system’s steady state in closed form, providing insights into the fundamental mechanisms underlying the observed dynamics. The detailed derivation of the model’s fixed points, which represent the equilibrium states, is provided in *SI Appendix*, Text 1. Readers are also referred to *SI Appendix*, Text 9 for an extended analysis using a complex, risk-stratified model for STI spread.

### Central Epidemiological Variables That Can Be Observed.

Given the modeling nature of our study, we can determine the exact fraction of the population infected (and infectious) with an STI-i.e., the total prevalence-at any point in time. In reality, however, prevalence can only be estimated through testing. To account for this, we introduce N and $N^{\text{obs}}$. The true prevalence, N, represents the total number of active infections, whereas the observed cases, $N^{\text{obs}}$-i.e., the number of positive test results-depend on the testing rates $\lambda_{s}$ and $\lambda_{a}$, as well as on N.

[10] $$\begin{array}{cccc} N & =I^{s}+I^{a} & :\; & \text{Total prevalence}\text{(active cases),} \end{array}$$

[11] $$\begin{array}{cccc} N^{\text{obs}} & =\lambda_{s}I^{s}+\lambda_{a}I^{a} & :\; & \text{Daily positive tests.} \end{array}$$

## Results

### Increasing PrEP Uptake Can Reduce the Prevalence of CT When Accompanied by Sufficiently Frequent Screening.

Increasing the uptake of PrEP, despite its association with reduced condom use, can effectively lower the prevalence of CT and other curable STIs among high-infection-risk groups when accompanied by sufficiently frequent, PrEP-related screening. This seemingly counterintuitive outcome arises because frequent screening enables the early detection and treatment of infectious but asymptomatic individuals, thereby reducing the time they contribute to disease transmission. Testing, as an active intervention, decreases the effective force of infection and lowers the overall reproduction number, potentially bringing it below one (Tables 1 and 2).

**Table 2.** Model parameters

Parameter	Definition	Value	Units	Source
$\beta_{0}^{\text{HIV}}$	Base spreading rate of HIV within risk group	0.6341	yr-1	(3)
$\beta_{0}^{\text{STI}}$	Base spreading rate of CT within risk group	{0.008,0.0112}	day-1	(45)
γ	CT recovery rate (natural)	1/1.32	yr-1	(46)
$\tilde{\gamma }$	CT treatment-mediated recovery rate	1/7	day-1	(46)
Σ	Total influx people infected with STI	0.01	yr-1	Assumed
ψ	Fraction of asymptomatic CT infection	0.85	-	(47, 48)
Φ	Recruitment rate to sexually active population	1/45	yr-1	(3)
μ	Exit rate from sexually active population	1/45	yr-1	(3)
$l_{\text{STI}}$	Incubation period for CT infection in men	14	day	(48)
$\lambda_{P}$	PrEP-related screening rate for STI	[0,4]	yr-1	(49, 50), scanned
$k_{\text{or}}$	Odds ratio of perceiving risk if one is at risk	50	-	(51)
$m_{\text{min}}$	Minimum mitigation	0	-	Assumed
$m_{\text{max}}$	Maximum mitigation	1	-	Assumed
$H_{\text{max}}$	Characteristic reaction awareness	0.2	-	Assumed
ξ	Risk assimilation	0	-	Assumed

**Fig. 2.**
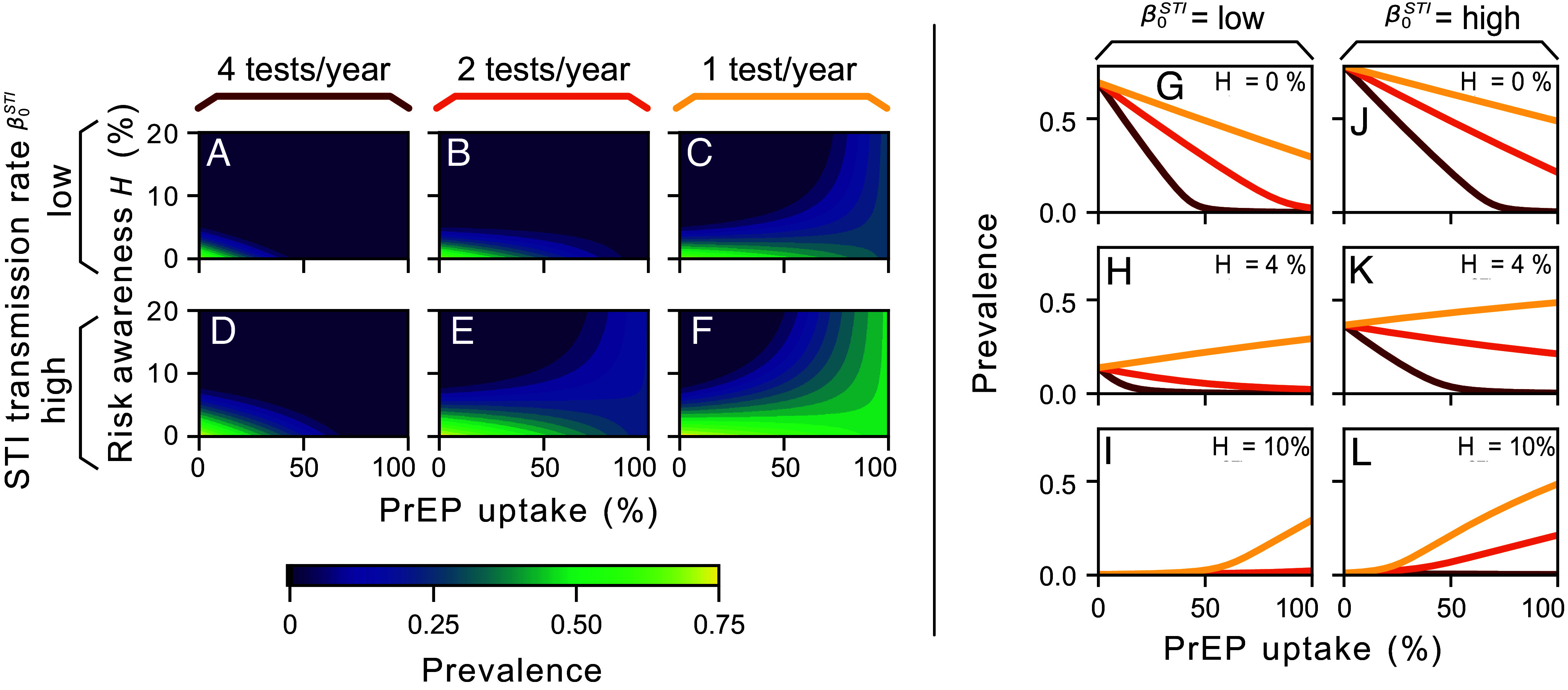
Increasing PrEP uptake among high-infection-risk groups can mitigate the spread of CT if asymptomatic screening is frequent enough. (*A*–*F*) We analyze the expected prevalence of CT infections under two scenarios of transmission rates (low and high; parameters in Table 2). The frequency of PrEP-related screening determines the effects of PrEP uptake, particularly at low levels of risk awareness in the population (H). For instance, when PrEP users are required to undergo STI screening every three months (*A* and *D*), higher PrEP uptake effectively reduces the expected prevalence. This benefit diminishes as screening frequency decreases. (*G*–*L*) The relationship between risk awareness and PrEP uptake is complex; increasing PrEP uptake results in a reduction of the fraction of the population that is sensitive to H and thereby reduces the relative impact of their self-protective actions. At low risk awareness, PrEP screening dominates, and the overall effect of PrEP is beneficial (*G*), but this effect can reverse when awareness is higher (*H* and *I*). This effect is more pronounced when the STI transmission rate is high (*J*–*L*).

We analyze two scenarios regarding the transmission rate of CT alongside three different (PrEP-related) screening frequencies within high-infection-risk groups of MSM (Fig. 2). At high screening frequencies (i.e., 4 tests per year), increased PrEP uptake correlates with a reduction in CT prevalence. However, with decreasing screening frequency, a larger increase in PrEP uptake is necessary to prevent an increase in prevalence. Furthermore, if the screening frequency falls below a critical threshold, the net effect of PrEP reverses to be detrimental—asymptomatic screening is not sufficient to counteract the increased spreading rate caused by the drop in condom use among PrEP users (Fig. 2*A*–*F* and *SI Appendix*, Fig. S1 *A*–*F* and Text 2).

The relationship between risk awareness and PrEP uptake is complex; increasing PrEP uptake results in a reduction of the fraction of the population that is sensitive to H and thereby reduces the relative impact of their self-protective actions. On the one hand, at very low risk awareness (i.e., when H≈0), the overall asymptomatic STI screening is dominated by the PrEP-related testing, even reaching the elimination threshold when a critical threshold of PrEP uptake is reached. However, this critical threshold is higher when screening occurs less frequently, which could render it unachievable if screening rates fall below a critical point (cf. to Fig. 2*G*). On the other hand, when H>0%, the effect of the risk-perception-driven testing $\lambda_{H}$ may dominate the overall testing rates, thus requiring more frequent PrEP-related screening $\lambda_{P}$ to compensate in situations where PrEP use is widespread. In other words, larger PrEP uptake leads to higher CT prevalence in situations where PrEP-related screening is low (Fig. 2 *H* and *I* and *SI Appendix*, Fig. S1). This highlights the potential of frequent STI screening for PrEP users, together with high levels of risk awareness, to decrease STI prevalence.

Importantly, even for a lower fraction of asymptomatic infections [ψ=0.7 (52)], the dynamics remain consistent: Prevalence increases with PrEP uptake when PrEP-related testing is low and decreases when testing is frequent (*SI Appendix*, Fig. S2). The overall prevalence, however, decreases. This is because individuals with symptoms will voluntarily seek treatment and are thus removed from the infection pool. Asymptomatic screening, therefore, plays a smaller role, making the system less reliant on frequent asymptomatic testing to interrupt transmission. Additional analysis of testing frequencies, including 0, 3, and 5 tests per year (*SI Appendix*, Fig. S3), is provided in *SI Appendix*, Text 4, further supporting the robustness of our conclusions across a range of testing scenarios.

### Testing Paradox: Observing More but Having Less.

**Fig. 3.**
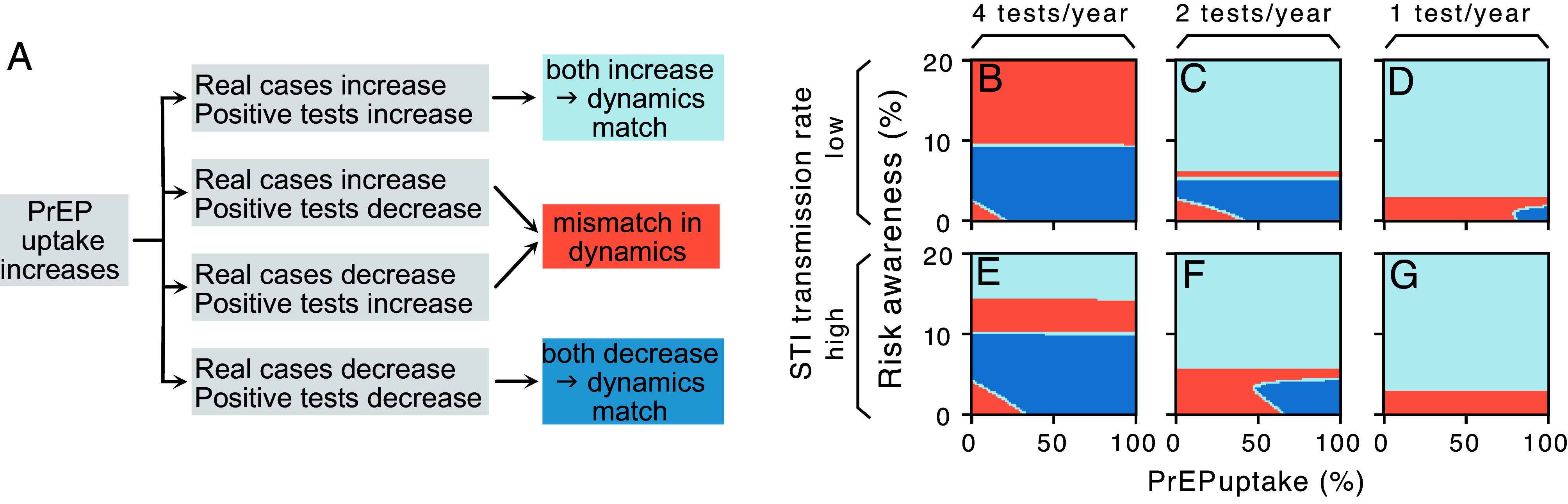
Mismatch in the dynamics of observed and real cases. (*A*) An increase in PrEP uptake will change the prevalence N and observed cases $N^{\text{obs}}$. Both can either increase or decrease, resulting in four different combinations that can be assigned into three categories: (light blue) both N and $N^{\text{obs}}$ will increase as a consequence of increased PrEP uptake in the population, (dark blue) both N and $N^{\text{obs}}$ will decrease as a consequence of increased PrEP uptake in the population, (red) one of the two increases, while the other decreases. This last scenario leads to a mismatch in dynamics. This means that the observed dynamics (number of positive tests) do not represent the true trends of STI prevalence and thus give a false picture of what is happening. (*B*–*G*) Computing the product of the derivatives of N and $N^{\text{obs}}$ with respect to PrEP uptake ($\frac{\partial N}{\partial P}\cdot \frac{\partial N^{\text{obs}}}{\partial P}$) unveils whether the change in observed cases matches the change in real cases. We found several regions with mismatches (red) where real cases decrease while observed cases rise. (Parameters in Table 2).

When analyzing how PrEP uptake and risk awareness in the population affect CT spread, we must distinguish between true prevalence (N; Eq. **10**) and observed cases (number of positive tests, $N^{\text{obs}}$; Eq. **11**). Typically, we assume that while $N^{\text{obs}}$ underestimates N, it is a reliable signal of its trends. However, while more frequent screening increases the number of cases uncovered, it also reduces the true prevalence, as these uncovered individuals are treated and do not contribute to spreading the disease further. This creates a paradoxical result; we may see more cases while the true prevalence declines. To understand when and why this paradox arises, we analyzed the rates of change in N and $N^{\text{obs}}$ with respect to increasing PrEP uptake (Fig. 3), i.e., how much do they change when increasing a unit of PrEP uptake. These rates are mathematically quantified by their partial derivatives ($\frac{\partial N}{\partial P}$, $\frac{\partial N^{\text{obs}}}{\partial P}$). For a wide range of parameters, these derivatives have matching signs. Therefore, $N^{\text{obs}}$ is a reliable signal for the direction of changes in N (i.e., both increase or decrease together, respectively light and dark blue in Fig. 3; also see *SI Appendix*, Fig. S6 and Text 6). Regions where the product of the partial derivatives of N and $N^{\text{obs}}$ is negative (red) are where the paradox arises: One quantity increases (positive derivative) and the other decreases (negative derivative).

The contribution of PrEP to the overall testing rate goes beyond the mandatory screening: As a consequence of risk compensation among PrEP users, PrEP uptake shapes the testing behavior in the whole population. When increasing PrEP uptake, the fraction of the population that is not on PrEP (and thus reacts to the perceived HIV infection risk) decreases. This can lower the overall STI testing rate, especially when PrEP-related testing rates $\lambda_{P}$ are low (*SI Appendix*, Fig. S7 and Text 7). When the overall testing increases, previously undiscovered infections become uncovered, and people receive treatment. If there are more susceptible than infectious individuals (i.e., S>I), the force of infection decreases, thereby reducing N. For the dynamics of $N^{\text{obs}}$, two factors play a role: If N stays constant, increased testing leads to higher $N^{\text{obs}}$ simply because we test more. If the testing rate stays constant, on the other hand, an increase or decrease in N will lead to an increase or decrease in $N^{\text{obs}}$, respectively, because the positivity rate changes. When both the positivity rate and the overall number of tests change simultaneously, the change in $N^{\text{obs}}$ depends on which of the two has the bigger effect.

If risk awareness and PrEP uptake both are low, increasing PrEP uptake will increase the overall testing rate, causing N to decrease and $N^{\text{obs}}$ to increase (Lower Left corners in Fig. 3*B*–*F*). On the other hand, increasing PrEP uptake at low risk awareness and high PrEP uptake will most likely increase the overall testing rate so that N decreases fast enough to cause a decline in $N^{\text{obs}}$ too. This effect is more pronounced when the PrEP-related screening rate is high. However, for medium risk awareness, risk-related and PrEP-related testing frequencies are similar, thus the overall testing increases only by a small amount as PrEP uptake increases. Consequently, N, and therefore the positivity rate, also only decreases by a small amount. The overall increase in testing, therefore, has a larger effect and leads to an increase in $N^{\text{obs}}$. With higher PrEP-related testing frequency, this region starts at higher risk awareness levels and spans larger ranges of risk awareness (Fig. 3 *B* and *E*). For lower testing rates, on the other hand, risk-related testing is higher than PrEP-related testing even at relatively low risk awareness levels, so that increasing PrEP uptake means less total testing, leading to an increase in both real and observed cases (Fig. 3*C*, *D*, *F*, and *G*). Even though we do not observe it here, it could also happen that N increases while $N^{\text{obs}}$ decreases. This would be the case if testing decreases at fast levels, fueling new infections while at the same time observing fewer simply because we test less (*SI Appendix*, Fig. S4). Increasing PrEP uptake in the population can therefore have differing effects on N and $N^{\text{obs}}$ depending on multiple factors, including risk awareness and PrEP-related testing frequency.

Another quantity of epidemiological importance is the positivity rate, defined as the proportion of tests that are positive on a given day. Since the positivity rate follows the same dynamics as N, the paradox does not emerge (*SI Appendix*, Fig. S6). Although this would intuitively be a better indicator of the true dynamics, the applicability of this variable is limited: To accurately determine the positivity rate, one must know the total number of tests conducted on that day, including both positive and negative results.

Note that all analyses in this section, which focused on changing PrEP uptake in the population, can also be done with respect to changing risk awareness. This analysis, alongside robustness tests, can be found in *SI Appendix*, Text 5.

## Discussion

Our analysis revealed that PrEP-related testing can cause paradoxical trends in observed STI incidence when PrEP uptake or risk awareness increases within the population. As testing becomes more frequent, surveillance data may suggest a rise in STI incidence (observed) in situations where the actual trends are declining. This apparent contradiction arises from the combined effects of risk- and PrEP-related testing as well as risk compensation. Importantly, the conditions that give rise to this paradox are realistic: Both PrEP uptake and risk awareness vary considerably across countries and communities, making it likely that such dynamics may occur in real life rather than being a purely theoretical artifact. In fact, our findings provide a causal path to reconcile conflicting observational data on the effects of PrEP (15, 18–23, 26, 53, 54).

Aligned with other modeling studies (29), our model suggests that increasing PrEP uptake can help to reduce the prevalence of CT in high-infection-risk groups of MSM, but only if asymptomatic PrEP-related mandatory testing is sufficient. Without frequent screening, testing cannot counteract the risk compensation among PrEP users, and the secondary benefits of PrEP in controlling CT prevalence may be reversed. Thus, regular and frequent screening should remain a fundamental part of PrEP guidelines, as it is in several European countries (32).

However, there is some debate in the field related to the benefits of frequent asymptomatic screening for some STIs (52), especially for CT and NG, arguing that there is no clear link between screening and reduced rates for these STIs among MSM (55), and it may cause an increased use of broad-spectrum antibiotics (56). First, observational data show conflicting evidence on whether more screening led to permanent reductions in the incidence of CT and NG (55, 57). The mechanism we identified reconciles these results, demonstrating that across a broad range of parameters, testing artifacts account for the observed rise in STI prevalence, even as the true prevalence declines. Second, although more frequent screening indeed leads to overexposure to antibiotics (57), we stress that this is not a consequence of asymptomatic screening but rather a lack of personalization in treating such infections: Alternative and personalized antimicrobial regimens limiting the use of azithromycin should also be considered (58). Furthermore, the behavioral component of knowing one’s health status is independent of the availability of a treatment: Once one is aware of being infectious, reacting to protect others bears no or only negligible costs to the person (59, 60).

Our model includes several simplifying assumptions, made to isolate key mechanisms and facilitate the interpretation of the dynamics. First, we assume that risk awareness is proportional to HIV prevalence and remains constant over time, justified by a separation of timescales: HIV spreads much more slowly than curable STIs like CT. Following this argument, we assume no functional or causal relationship between PrEP uptake and risk awareness. Instead, we consider multiple combinations of these two variables (PrEP uptake from 0 to 100% and risk awareness up to 20%), treating them as independent for the purpose of our analysis. These values are chosen to reflect values that correspond to plausible real-world scenarios (61–63), given the way risk awareness influences behavior in the model. Our modeling approach and parameterization are particularly relevant for countries with well-established PrEP programs and frequent STI screening practices among MSM. Many countries already recommend regular PrEP-related testing, though it often is not mandatory (64–67). Conversely, our framework is less directly applicable to settings where PrEP coverage is low, testing infrastructure is limited, or epidemiological data specific to MSM are sparse, such as in many low- and middle-income countries.

To ensure our findings also hold for more realistic modeling choices, we also explored a more complex version of the model that stratifies the population into four validated infection-risk groups (*SI Appendix*, Text 9). In this extended framework, individuals differ in their levels of risk awareness, infection risk, and contact patterns between groups—features that more closely reflect real-world heterogeneity. Despite the substantial increase in complexity, results remain qualitatively consistent (cf. *SI Appendix*, Fig. S10 and S12 to mirror Fig. 2 and *SI Appendix*, Fig. S11 and S13 to mirror Fig. 3). This suggests that the core insights derived from the minimal model are robust and general.

To sum up, the effectiveness of interventions like PrEP needs to be carefully assessed to rule out testing artifacts, as they affect both the dynamics and screening rates of STIs. We showed a plausible mechanism to reconcile conflicting observational evidence on the effects of PrEP on the prevalence of STIs, together with analyzing the sensitivity of such effects with regard to different levels of risk awareness, PrEP-related testing frequencies, and transmission rates. Wherever resources are available, asymptomatic screening together with personalized treatment of infections remains an attractive way to slow down the spread of STIs, including HIV (29, 68), especially in high-infection-risk groups. Individuals in these groups may act as bridges to other populations and settings where PrEP uptake is not widespread. Ultimately, increasing the prevalence of CT and other curable STIs in these unprotected groups increases their susceptibility to acquiring HIV.

## Supplementary Material

Appendix 01 (PDF)

## Data Availability

All code to reproduce the analysis and figures shown in the manuscript as well as in *SI Appendix* is available online on GitHub https://github.com/Priesemann-Group/testing-paradox-HIV-STI (69). All other data are included in the manuscript and/or *SI Appendix*.
