# Supplementary material for: Testing paradox may explain increased observed prevalence of bacterial STIs among MSM on HIV PrEP: A modeling study
Source: Proc Natl Acad Sci U S A. 2025 Oct 29;122(44):e2524944122. doi: 10.1073/pnas.2524944122 (PMC12595453; doi:10.1073/pnas.2524944122)
Supplement: Supplementary file 1 — Appendix 01 (PDF) [file pnas.2524944122.sapp.pdf]

PNAS

Supporting Information for

Testing Paradox May Explain Increased Observed Prevalence of Bacterial STIs among MSM on HIV PrEP: A Modeling Study

Laura Müller, Piklu Mallick, Antonio B. Marín-Carballo, Philipp Dönges, Robyn J. N. Kettlitz, Carolina J. Klett-Tammen, Mirjam Kretzschmar, Viola Priesemann and Seba Contreras

Seba Contreras.
E-mail: seba.contreras@ds.mpg.de

This PDF file includes:

Supporting text
Figs. S1 to S13
Table S1
SI References

Supporting Information Text

Supporting Information Text 1: Fixed points of the simple HIV-STI model. In our model, the population is divided into compartments corresponding to different health and disease states: susceptible (S), asymptomatic infectious (I^a), symptomatic infectious (I^s), and on treatment (T). Each compartment represents the fraction of the total population in that category. We assume the total population remains constant over time and normalize it to 1:

$$S + I^a + I^s + T = 1. \tag{1}$$

This condition, known as demographic equilibrium, implies that the sum of all derivatives is zero. This allows us to obtain an expression for the recruitment rate Φ:

$$\Phi = \mu\,(S + I^a + I^s + T) = \mu. \tag{2}$$

Additionally, it allows reducing the system to three differential equations, given that T can be represented as an algebraic function of the other variables:

$$T = 1 - (S + I^a + I^s)\,. \tag{3}$$

We determine the existence of fixed points (S^*, I_a^*, I_s^*) by imposing the stability condition $\frac{d}{dt}\left(S^*, I_a^*, I_s^*\right) = (0,0,0)$. Substituting the conditions above, Eq. 1 – 3 in the main text can be rewritten as follows:

$$0 = -C_1\left(I_a^* + I_s^*\right)S^* + \gamma I_a^* + \tilde{\gamma}\left(1 - (S^* + I_a^* + I_s^*)\right) + \mu - \mu S^* - \Sigma\,, \tag{4}$$

$$0 = \psi C_1\left(I_a^* + I_s^*\right)S^* - (\gamma + \lambda_a)I_a^* - \mu I_a^* + \psi\Sigma\,, \tag{5}$$

$$0 = (1-\psi)C_1\left(I_a^* + I_s^*\right)S^* - (\lambda_s + \mu)I_s^* + (1-\psi)\Sigma\,, \tag{6}$$

where $C_1 = \beta_0^{\mathrm{STI}}\left(1 - m(H)\,(1-P)\right)$.

We calculate S^* from equation Eq. (6):

$$S^* = \frac{(\lambda_s + \mu)I_s^* - (1-\psi)\Sigma}{(1-\psi)C_1(I_a^* + I_s^*)}\,. \tag{7}$$

The relationship between I_s^* and I_a^* follows from equations Eq. (5) and Eq. (7):

$$I_s^* = \frac{(\gamma + \lambda_a + \mu)(1-\psi)}{\psi(\lambda_s + \mu)} I_a^* \overset{\mathrm{def}}{=} C_2 I_a^*\,. \tag{8}$$

Using equations Eq. (4), Eq. (7), and Eq. (8), we obtain a quadratic equation for I_a^*

$$a(I_a^*)^2 + bI_a^* + c = 0\,, \tag{9}$$

where

$$a = C_1(1 + C_2)\left[-(\lambda_s + \mu)C_2 + (\gamma - \tilde{\gamma}(1 + C_2))(1-\psi)\right]\,, \tag{10}$$

$$b = (\tilde{\gamma} + \mu)\left[C_1(1-\psi)(1+C_2) - (\lambda_s + \mu)C_2\right]\,, \tag{11}$$

$$c = (\tilde{\gamma} + \mu)(1-\psi)\Sigma\,. \tag{12}$$

$$\tag{13}$$

From this follows:

$$I_a^* = \frac{-b \pm \sqrt{b^2 - 4ac}}{2a}\,. \tag{14}$$

Given that C_1, $C_2 > 0$, and assuming that $\tilde{\gamma}\,(1 + C_2) > \gamma$ (which in the parameter regime we explore is true even for the case $C_2 = 0$), we can conclude that:

- In Eq. 9, $a < 0$ and $c > 0$.
- Both solutions are real, given that $b^2 - 4ac > 0$ always.
- There is only one positive solution for I_a^* in Eq. 14, given that the product of the roots of Eq. 9 fulfills $x_1 x_2 = c/a < 0$.

We therefore showed the existence of one positive real-valued fixed point.

Supporting Information Text 2: Minimum PrEP-related testing frequency. We demonstrated that insufficient PrEP-related testing leads to an increase in STI prevalence as PrEP uptake increases. This led us to question whether a minimum PrEP-related testing frequency, $\lambda_{P,\min}$, exists, which is necessary to decrease STI prevalence. The parameter λ_P represents the number of mandatory tests per year for individuals taking PrEP. In the main analysis, we considered three specific values (1, 2, and 4 tests per year). Here, we adopt a more qualitative approach to explore whether a minimum required testing frequency, $\lambda_{P,\min}$, emerges as a function of PrEP uptake (P) and risk awareness (H).

For a fixed PrEP uptake P and risk awareness H, increasing the PrEP-related testing frequency λ_P consistently reduces STI prevalence, as expected (Fig. S1a-f). For example, for high STI transmission, $P = 65\,\%$, and $H = 0\,\%$, increasing λ_P from 1/year to 4/year decreases prevalence by 88.47 % (from 0.581 to 0.067). This suggests that more testing is always better when PrEP uptake and risk awareness are constant. However, when risk awareness H is moderate or high (here, $H \gtrsim 4$), insufficient PrEP-related testing leads to an increase in STI prevalence as PrEP uptake rises (Fig. S1b,c,e,f). This occurs because individuals on PrEP undergo mandatory PrEP-related testing but not additional risk-related testing. This means that when the PrEP-related testing frequency is lower than the risk-related testing frequency, overall testing decreases as more people adopt PrEP, driving up STI prevalence. From these observations, we conclude that a minimum PrEP-related testing frequency, $\lambda_{P,\min}$, exists and depends on risk awareness.

In summary, insufficient PrEP-related testing frequency can contribute to a rise in STI prevalence as PrEP uptake in the population increases. Consequently, a minimum testing frequency, $\lambda_{P,\min}$, exists, which depends on risk awareness. However, testing above this threshold is always beneficial and leads to a further reduction in STI prevalence.

Supporting Information Text 3: Impact of asymptomatic fraction on disease dynamics. In the main text, we used an asymptomatic fraction of $\psi = 0.85$ (1, 2) (see Table 1 in the main text). To assess the impact of this parameter, we performed a sensitivity analysis in which the asymptomatic fraction was reduced to 0.7, following the value suggested by William *et al.*(3).

Our simulations indicate that this reduction in the asymptomatic fraction does not lead to any qualitative changes in the overall epidemic dynamics; the trends observed in the main analysis remain consistent (Fig. S2). However, as expected, a lower asymptomatic fraction decreases overall prevalence compared to the baseline scenario (cf. Fig. 2 in the main text). This outcome is not surprising; individuals are assumed to seek testing and treatment voluntarily as they develop symptoms, removing themselves earlier from the pool of infection should that be the case.

Supporting Information Text 4: Results for additional PrEP-related testing frequencies. We tested how sensitive our results are to the rate of PrEP-related screening. Keeping all other parameters at their baseline values, we repeated the analysis for $\lambda_P = 0$, 3, and 5 (Fig. S3, S4).

When there is no PrEP-related screening (0 tests per year), prevalence increases with PrEP uptake, consistent with the pattern seen under low testing in the main text. The observed prevalence, however, decreases in some parameter regimes, leading to a paradox again, but in the opposite direction as before: Now, the actual prevalence increases while the observed one decreases. With more frequent screening, the rise in prevalence with increasing PrEP uptake is much smaller and often shifts to a decline as uptake grows. At 5 tests per year, prevalence declines with uptake over most of the range. This means that the original testing paradox, that the real prevalence decreases while the observed cases increase, appears again, similar to $\lambda_P = 2$ and 4.

These results are consistent with the scenarios shown in the main paper for 1, 2, and 4 tests per year. They show that our conclusions do not depend on a specific choice of testing frequency and that the most informative dynamics arise at non-zero testing rates. In practical terms, frequent PrEP-related screening helps ensure that higher PrEP uptake does not increase STI prevalence and can even lead to lower prevalence.

Supporting Information Text 5: Testing artifacts with rising risk awareness. In the main text, we looked at the changing dynamics of STI prevalence N and positive tests N^{obs} when PrEP uptake changes. Here, we will look at changing risk awareness in the population (Fig. S5).

The risk awareness represents how people perceive their risk of infection on average and undergo voluntary testing even without symptoms. Increasing risk awareness in the population, therefore, leads to increased testing and in our case lowers N. If both risk awareness and PrEP uptake are low, this increased testing decreases N at such a rate that N^{obs} can increase; although the fraction of positive tests declines, the overall volume of testing rises enough that the absolute number of positive results still increases. If PrEP uptake stays low and risk awareness increases further, however, both N^{obs} and N decrease. The real cases now decrease so much that even though we test more, fewer positive tests are recorded. When PrEP uptake is high, most people already undergo PrEP-related testing, and the increase in overall testing due to increased risk awareness is only small, so N^{obs} also decreases here. Only for a low PrEP-related testing rate and high transmission rate (Fig. S5f), N decreases so slowly that increased testing leads to increased N^{obs}; the paradoxical region has a larger extent, supporting the previous result that frequent testing for PrEP users is essential for effective containment. Overall, increasing risk awareness in the population has not only the positive effect of decreased N but also leads to N^{obs} correctly reflecting the decreasing trend of N.

Supporting Information Text 6: Comparison between absolute number of positive tests and positivity rate. In Fig. S6, we show the real prevalence, N, along with the number of positive tests, N^{obs}, and the positivity rate. The positivity rate is defined as the number of positive tests divided by the total number of tests. As N^{obs} does not take into account how many tests have been carried out in total, its dynamics do not always reflect those of N, meaning that the paradox arises. The positivity rate,

on the other hand, reflects the real dynamics. It is therefore a better indicator of STI prevalence. However, positivity rate is often not available as only positive tests get reported in many countries.

Supporting Information Text 7: Visualization of testing rates. The testing rate depends on both PrEP uptake and risk awareness. In Fig. S7, we show the total as well as asymptomatic testing rate as a function of varying PrEP uptake, risk awareness, and PrEP-related testing frequencies. For low PrEP-related testing ($\lambda_P = 1$), testing decreases with increasing PrEP uptake as PrEP-related testing is lower than risk-related testing; as people start taking PrEP, they test less than before. For high PrEP-related testing ($\lambda_P = 4$), on the other hand, testing increases with PrEP uptake as people on PrEP test more often than those who only test depending on risk.

Supporting Information Text 8: Partial mitigation of PrEP users ($\xi \neq 0$). In the main text, we considered the case $\xi = 0$, where ξ denotes the risk assimilation parameter, regulating the extent to which PrEP users engage in risk mitigation. A value of $\xi = 0$ corresponds to no mitigation at all. Here, we examine alternative values, namely $\xi = 0.2$, 0.6, and 1 (Fig. S8). When $\xi = 1$, PrEP users apply the same mitigation measures as individuals not on PrEP. We find that as ξ increases, the parameter space in which the paradox arises becomes smaller. For $\xi = 0.6$, which corresponds to the baseline scenario in Jenness et al. (4), the paradox still emerges across a notable range of parameters. Overall, as PrEP users adopt more mitigation measures, prevalence tends to decrease in parameter regimes where it previously increased, due to reduced transmission.

Supporting Information Text 9: Extended model with risk group stratification. While our main analysis is based on a homogeneous population model, we also considered an extended model with risk group stratification to better capture real-world heterogeneity (Fig. S9). This stratified model includes four distinct risk groups, with their relative sizes and interaction patterns informed by an HIV model for high-risk MSM using data from the Netherlands (5). The four risk groups comprise 45.1%, 35.3%, 12.5%, and 7.1% of the total MSM population, respectively (6). We denote the groups by indices $l \in \{1, 2, 3, 4\}$, ordered by increasing risk: group $l = 1$ represents individuals at the lowest risk, while group $l = 4$ includes those at the highest risk. Parameters that differ from the small model (Table 1 in the main text) are listed in Table S1.

$$
\begin{aligned}
\frac{dS_l}{dt} &= -\Lambda_l\, S_l + \gamma\, I_l^a + \tilde{\gamma} T_l + \Sigma + \Phi - \mu\, S_l, \\
\frac{dI_l^a}{dt} &= \psi\, \Lambda_l\, S_l - \gamma\, I_l^a - \lambda_{a,l}(P,H)\, I_l^a - \mu\, I_l^a + \psi\, \Sigma, \\
\frac{dI_l^s}{dt} &= (1-\psi)\, \Lambda_l\, S_l - \lambda_{s,l}(P,H)\, I_l^s - \mu\, I_l^s + (1-\psi)\, \Sigma, \\
\frac{dT_l}{dt} &= \lambda_{a,l}(P,H)\, I_l^a + \lambda_{s,l}(P,H)\, I_l^s - \tilde{\gamma} T_l - \mu\, T_l,
\end{aligned}
$$

where

$$
\begin{aligned}
\Lambda_l &= \beta_0^{STI} \left((1 - m(H))\ (1-P) + (1 - \xi_l\, m(H))\, P \right) \sum_{l'} M_{ll'} \frac{I_{l'}^a + I_{l'}^s}{N_{l'}}, \\
m(H) &= m_{\min} + (m_{\max} - m_{\min}) \left(1 - \exp\left(-\frac{H}{H_{\text{thres}}} \right) \right), \\
M_{ll'} &= \omega \frac{c_{l'}^{\text{HIV}}\, N_{l'}}{\sum_k c_k^{\text{HIV}}\, N_k} + (1-\omega) \delta_{ll'}, \\
\lambda_{a,l}(P,H) &= k_{\text{or},l}\, \beta_0^{HIV}\ (1 - m(H))\ H\, (1-P) + \lambda_P\, P, \\
\lambda_{s,l}(P,H) &= \lambda_0 + \lambda_a(P,H)\, .
\end{aligned}
$$

N_l is the population size in group l, and $\delta_{ll'} = 1$ if $l = l'$, otherwise $\delta_{ll'} = 0$.

The increased dimensionality and complexity introduced by risk group stratification made analytical calculation of the model's fixed points highly challenging. As a result, directly solving for equilibrium states was impractical.

To address this, we employed a numerical approach, simulating the model dynamics over a sufficient number of years to allow the system to evolve towards a stable equilibrium. Equilibrium was operationally defined as the point at which the values of all relevant state variables–namely, the fraction of susceptible (S_l), asymptomatically infected (I_l^a), symptomatically infected (I_l^s), and treated (T_l)–changed by less than a small threshold ϵ between consecutive time steps, i.e., $|x^{(t)} - x^{(t-1)}| \leq \epsilon$ for all $x \in \{S_l, I_l^a, I_l^s, T_l\}$. This stopping criterion ensured that the model output reflected steady-state conditions, minimizing the influence of initial conditions or transient dynamics. All results from the risk-stratified extended model, as presented in this study, were obtained from simulations in equilibrium.

Supporting Information Text 10: Sensitivity Analysis of the extended model. To assess the robustness of our model, we performed a sensitivity analysis in two parts. First, we varied the odds ratios (k_{or}) assigned to the different risk groups (l). Second, we examined alternative configurations of risk-group–stratified risk assimilation (ξ_l). Values for the different scenarios can be found in Tab. S1.

For the odds-ratio analysis, we considered three scenarios with distinct combinations of odds ratios for the four risk groups (7) (Fig. S10 and Fig. S11). To ensure comparability between scenarios, all sets of odds ratios were normalized such that the population-weighted mean odds ratio was fixed at $k_{\mathrm{or}} = 50$. This normalization isolates the effect of risk distribution while holding the average population risk constant.

For each scenario, we reproduced the main results (Fig. 2 in the main text and Fig. 3 in the main text) using the corresponding odds-ratio distributions. The absolute prevalence only shows minor deviations across the three scenarios; the overall qualitative dynamics and trends remained consistent with those presented in the main figures.

In the second part of the sensitivity analysis, we tested three different scenarios for risk-group–stratified risk assimilation ξ_l (Fig. S12 and Fig. S13). Again, the results showed no significant qualitative differences across these scenarios.

Table S1. Model parameters of the extended model with risk group stratification

Parameter	Definition	Value	Units	Source
ω	Mixing parameter	0.5	–	(5)
c_l^{HIV}	Average number of new partners per year in risk group l	$[0.13, 1.43, 5.44, 18.21]$	yr^{-1}	(5)
$k_{or,l}$	Odds ratio in risk group l, likelihood that one perceives risk if one is at risk	Scenario 1: $[50, 50, 50, 50]$<br>Scenario 2: $[2, 63.5, 100, 200]$<br>Scenario 3: $[14, 24, 167, 203]$	–	(7)
ξ_l	Risk assimilation in risk group l	Scenario 4: $[0, 0, 0, 0]$<br>Scenario 5: $[0.8, 0.6, 0.4, 0.2]$<br>Scenario 6: $[1, 0.6, 0.2, 0]$	–	Assumed

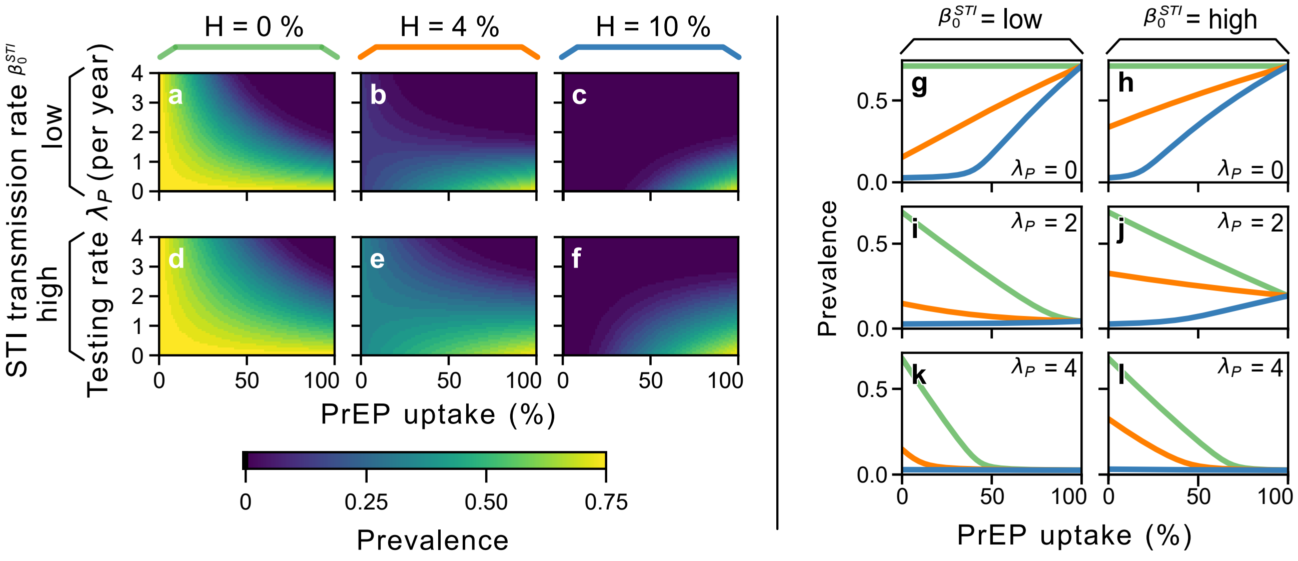

Fig. S1. Asymptomatic screening frequency among PrEP users is key to lowering STI prevalence. STI prevalence depends on the PrEP uptake, the PrEP-related testing rate λ_P, and risk awareness H (**a-f**). At high PrEP-related testing frequencies ($\lambda_P = 4$), STI prevalence never increases with increasing PrEP uptake for all levels of risk awareness (**a, d, k, l**). However, when PrEP-related testing frequencies λ_P is below the risk-related testing frequency, this relationship becomes non-monotonic at moderate or high risk awareness (here, $H \gtrapprox 4$), and more PrEP uptake can paradoxically lead to increased STI prevalence (**b, c, e, f, g-j**). This effect arises because PrEP users undergo mandatory PrEP-related testing only; thus, when λ_P is lower than the risk-related screening frequency, the overall screening decreases as PrEP uptake increases, resulting in higher STI prevalence. Parameters in Table 1 in the main text. Mirroring Fig. 2 in the main text.

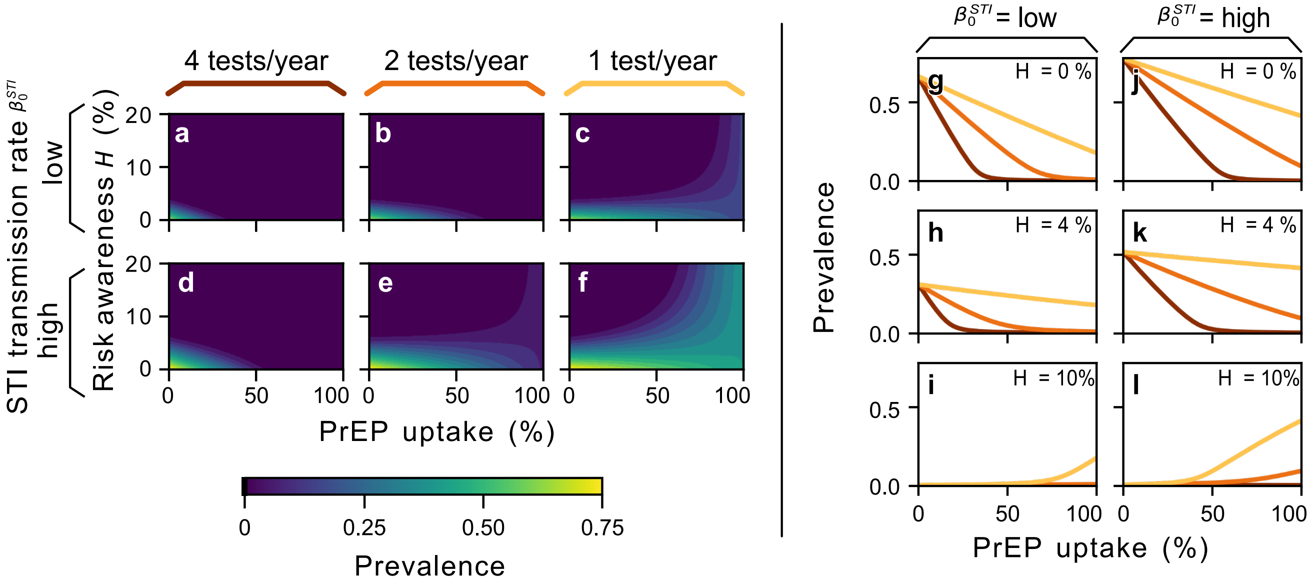

Fig. S2. Increasing PrEP uptake can mitigate the spread of chlamydia. We analyze the prevalence of chlamydia infections under two scenarios of transmission rates (low and high; parameters in Table 1 in the main text) for an asymptomatic fraction of $\psi = 0.7$ (in the main text we used $\psi = 0.85$). The frequency of PrEP-related testing determines the effects of PrEP uptake, particularly at low levels of risk awareness in the population (H). Mirroring Fig. 2 in the main text.

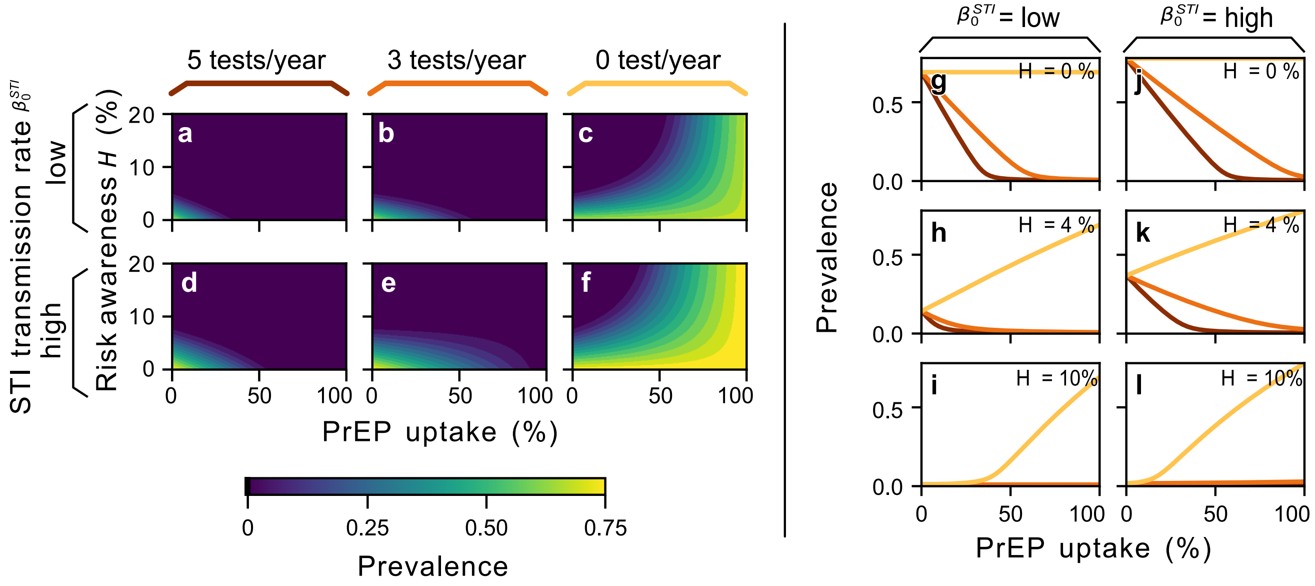

Fig. S3. Varying mandatory PrEP-related testing frequencies. Extension of Fig. 2 in the main text, showing scenarios with 0, 3, and 5 PrEP-related tests per year (instead of 1, 2, and 4).

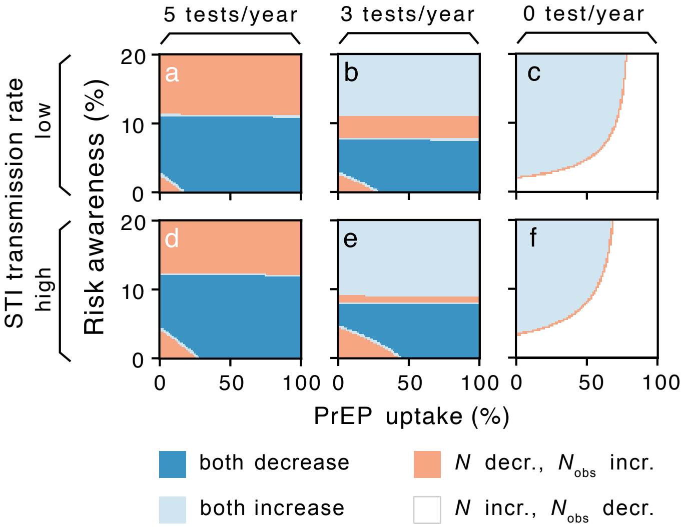

Fig. S4. Relation between real and observed dynamics for additional PrEP-related testing frequencies $\lambda_P = 0, 3, 5$. Mirroring Fig. 3 in the main text.

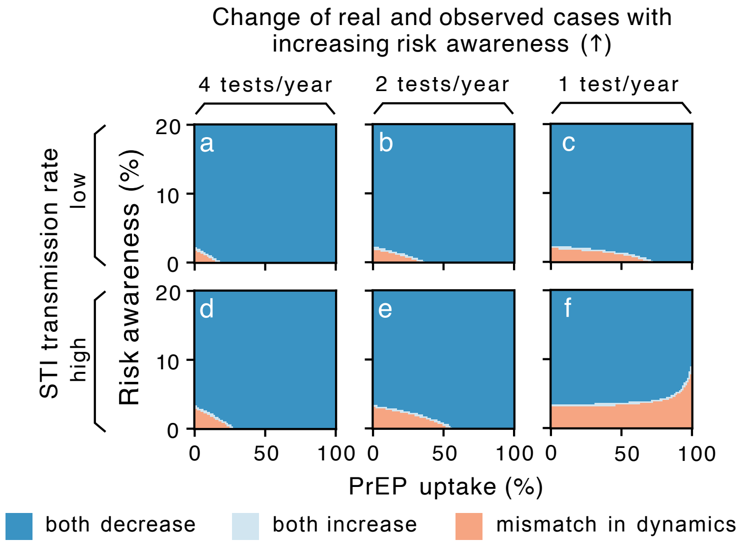

Fig. S5. Comparison of the dynamics of observed and real cases. An increase in risk awareness will change the prevalence N and observed cases N^{obs}. Both can either increase or decrease, resulting in four different combinations. These can be categorized into 3 categories: (light blue) both N and N^{obs} will increase, (dark blue) both N and N^{obs} will decrease, (red) one of the two increases, while the other decreases (in our case, we only observe the case where N decreases while N^{obs} increases). While the positive tests mirror the real dynamics most of the time, we found some regions with mismatches (red) where real cases decrease while observed cases rise. Parameters in Table 1 in the main text. Complementing Fig. 3 in the main text, where we looked at the change with PrEPuptake instead of risk awareness.

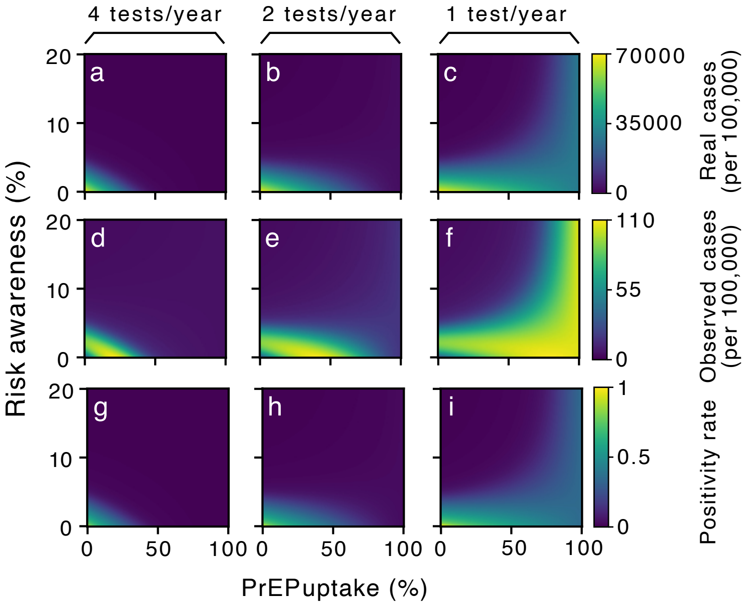

Fig. S6. Comparison between absolute number of positive tests and positivity rate. Observed positive tests (**d–f**) do not necessarily reflect the true number of STI cases (**a–c**). In contrast, the positivity rate—defined as the fraction of tests that are positive on a given day—appears to track the underlying dynamics more closely (**g–i**). However, this measure requires complete reporting of both positive and negative test results. This highlights the importance of comprehensive surveillance systems and detailed laboratory reporting, including both test results and reasons for testing, to minimize bias.

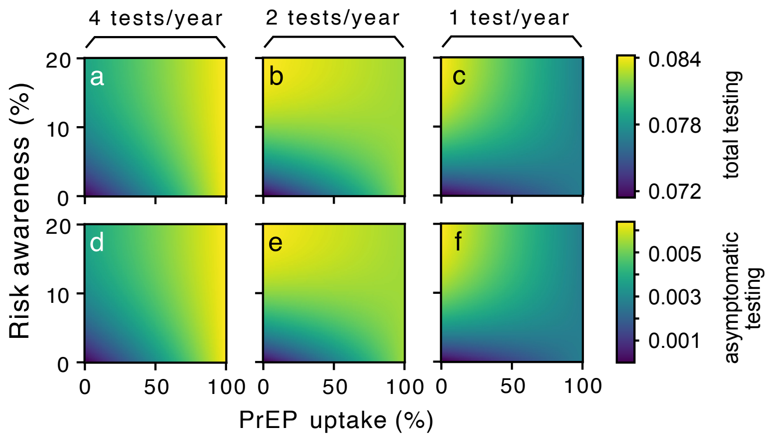

Fig. S7. Testing rates as a function of PrEP uptake and risk awareness. (**a-c**) Total testing, consisting of both symptomatic and asymptomatic testing. (**d-f**) Asymptomatic testing. For low PrEP-related testing frequency ($\lambda_P = 1$; **c,f**), testing mostly declines with increasing PrEP adoption as individuals taking PrEP test less as individuals not taking PrEP and only testing according to risk awareness. Only for low levels of risk awareness, testing increases with PrEP uptake as risk-related testing is minimal. For higher PrEP-related testing frequencies ($\lambda_P = 4$; **a,d**), however, testing increases with increasing PrEP uptake for all levels of risk awareness.

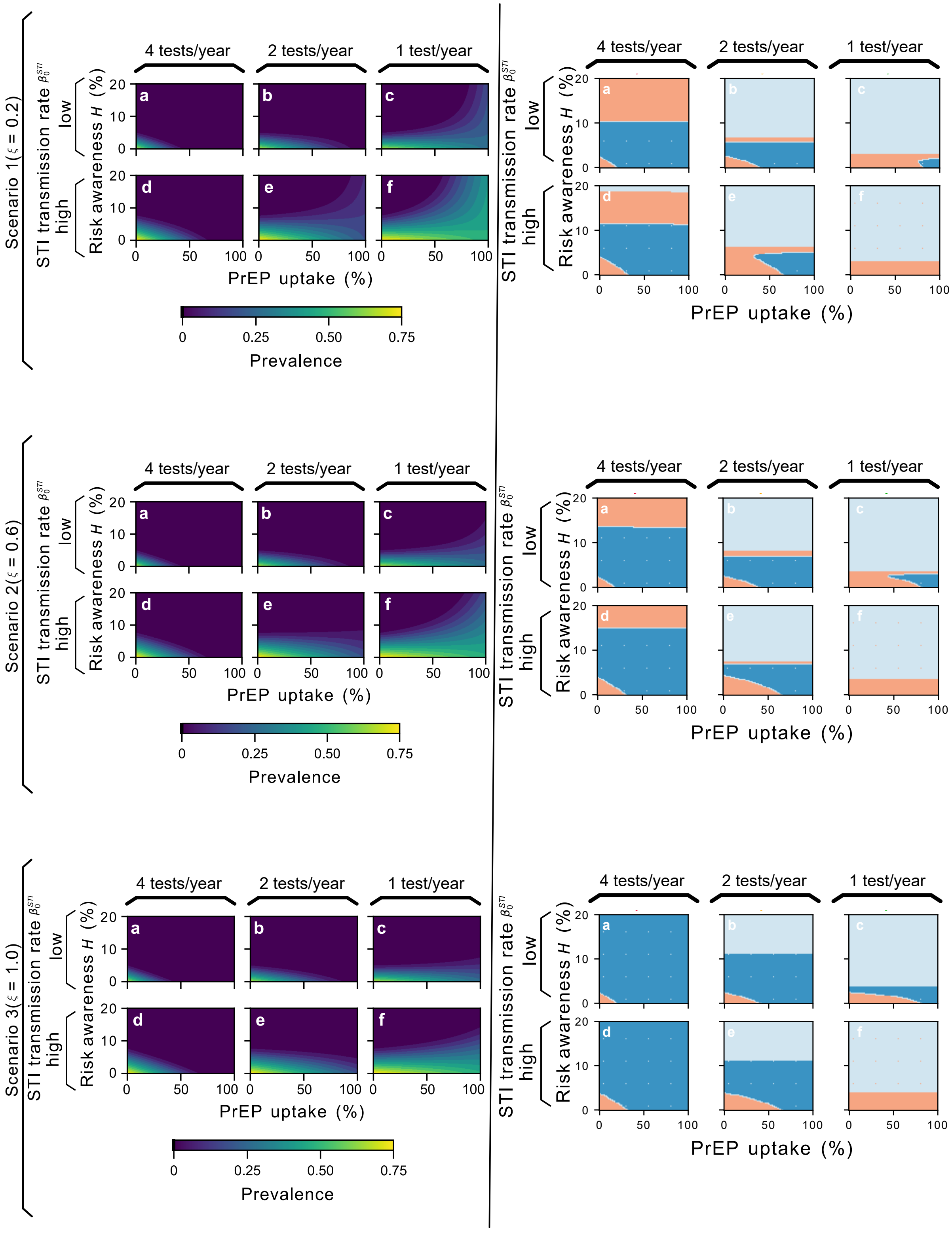

Fig. S8. Different levels of risk assimilation among PrEP users. The increasing trend of prevalence and the paradox exist even for different values of risk assimilation (for the exact value for each scenario see Tab. S1) (mirroring Fig. 2 in the main text and Fig. 3 in the main text)

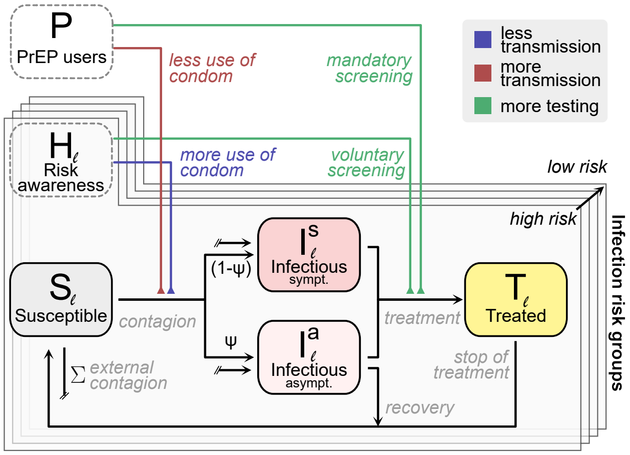

Fig. S9. Extended model for chlamydia transmission among high-risk MSM with risk-group stratification. This model extends the minimal model from the manuscript by dividing the high-risk MSM group into four risk groups, which differ, among other factors, in their number of partners, mitigation behavior and risk perception.

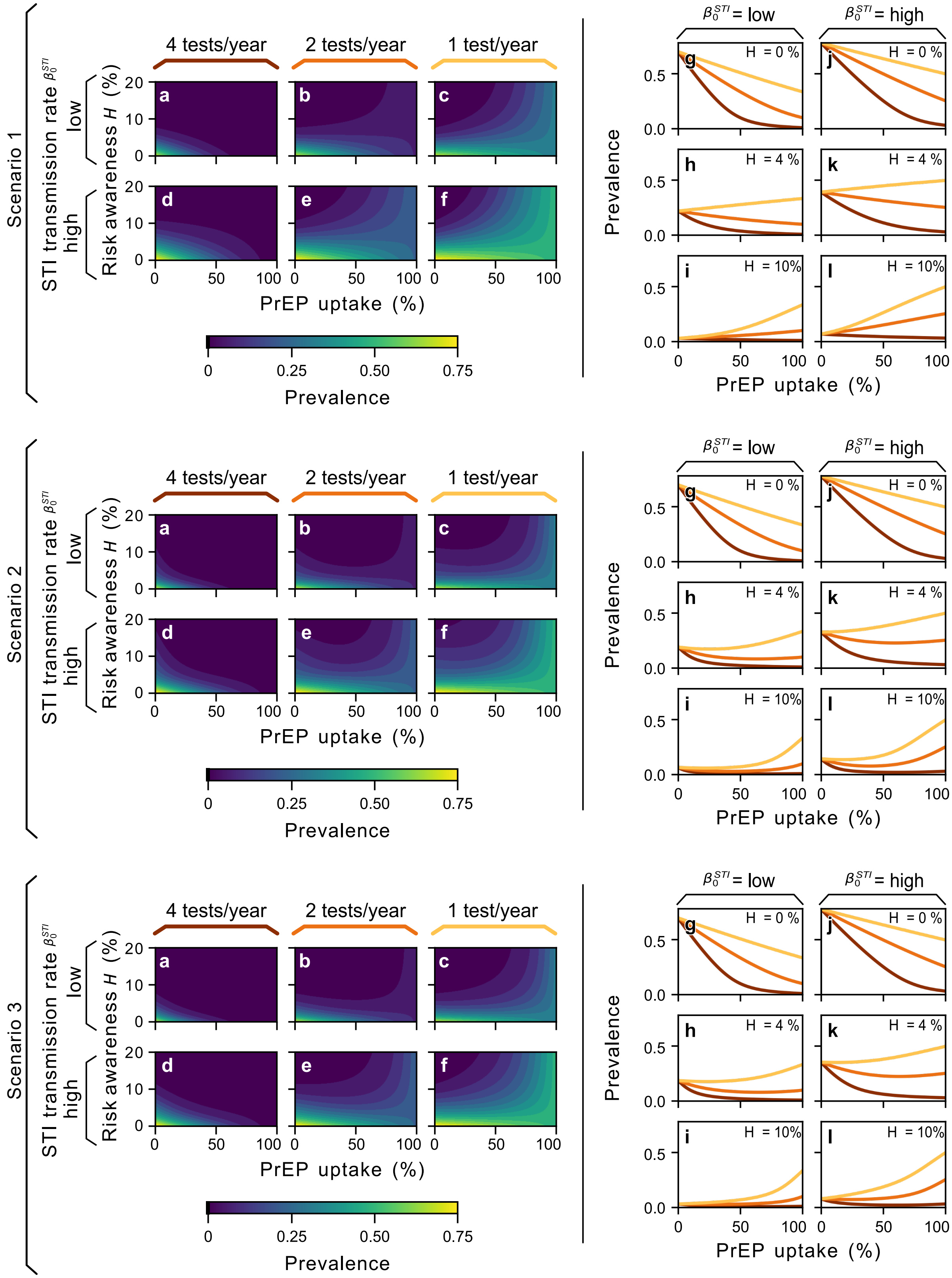

Fig. S10. Varying odds ratios among the risk groups in the extended model. For the exact value for each scenario, see Tab. S1. Mirroring Fig. 2 in the main text.

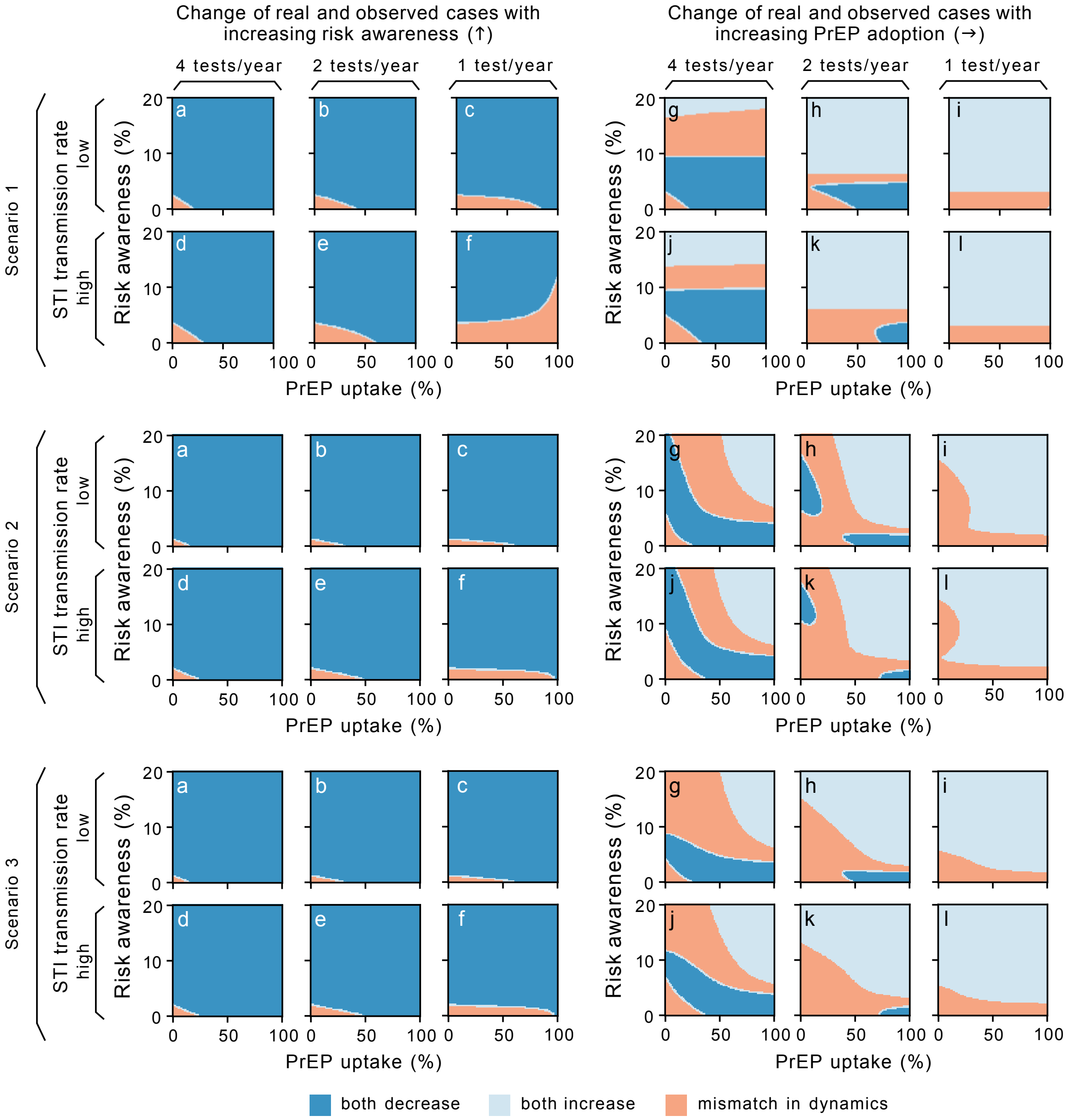

Fig. S11. Varying odds ratios among the risk groups in the extended model. For the exact value for each scenario, see Tab. S1. Mirroring Fig. 3 in the main text.

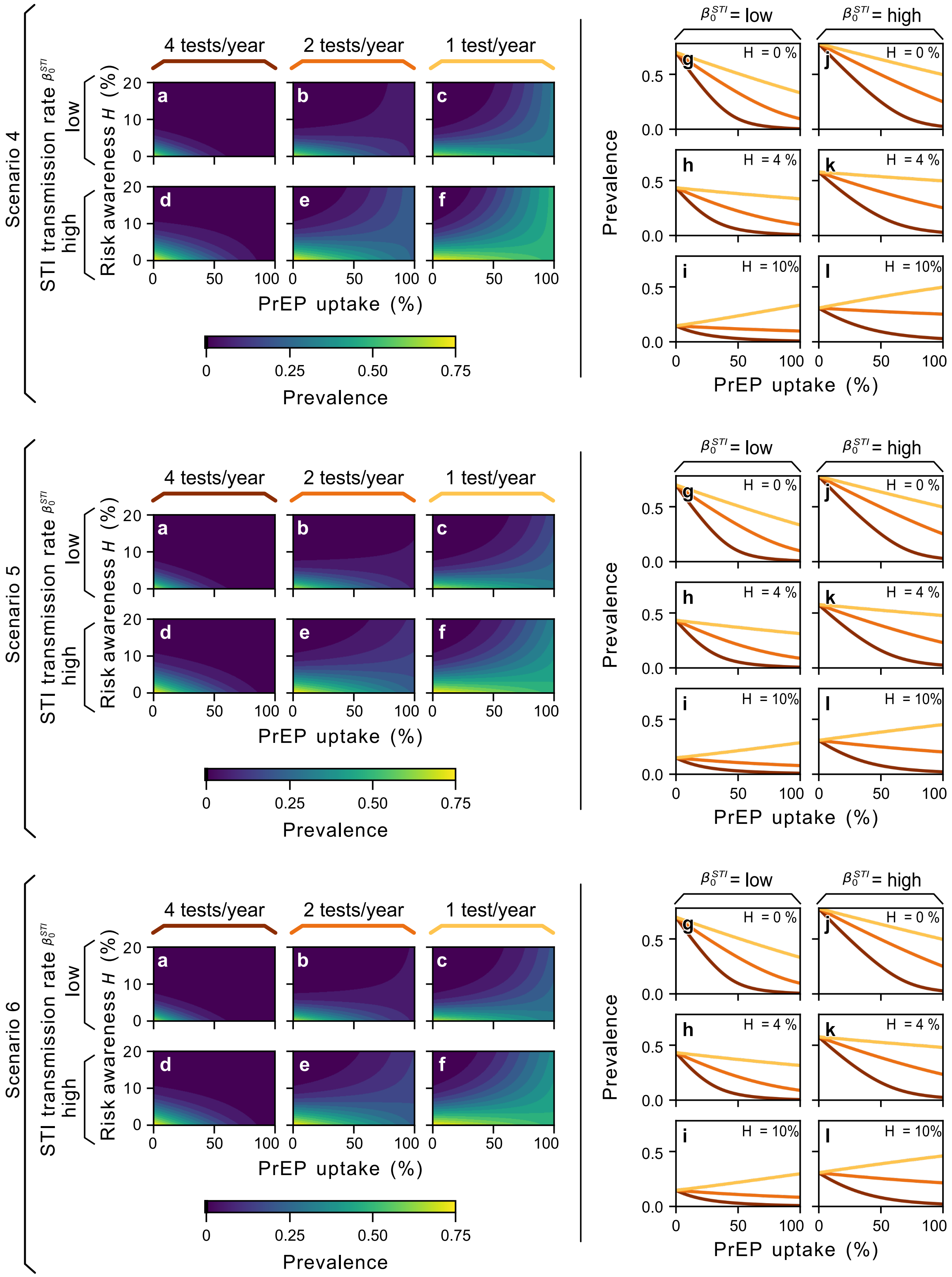

Fig. S12. Varying risk assimilation stratified by risk groups in the extended model. Three different scenarios with different combinations of ξ_l according to Tab. S1. Mirroring Fig. 2 in the main text.

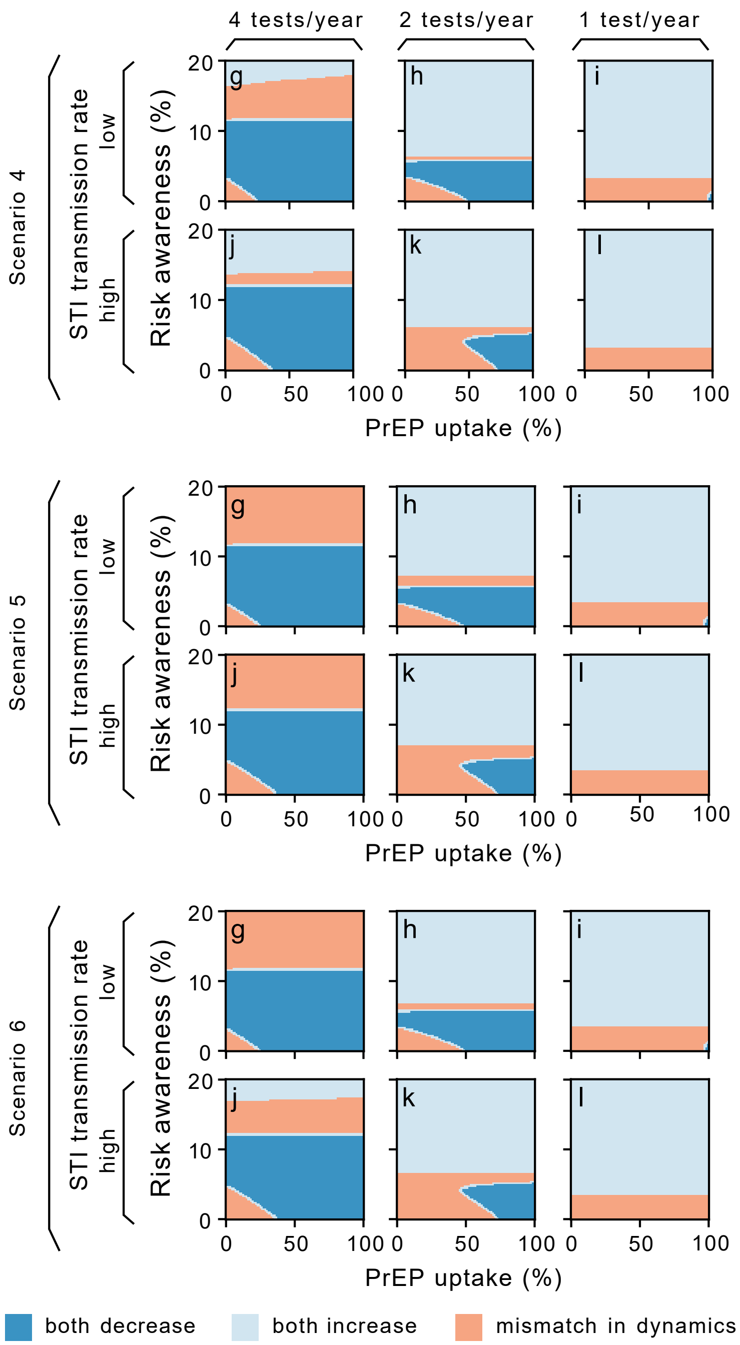

Fig. S13. Varying risk assimilation stratified by risk groups in the extended model. Three different scenarios with different combinations of ξ_l according to Tab. S1. Mirroring Fig. 3 in the main text.

References

1. DS LaMontagne, DN Fine, JM Marrazzo, Chlamydia trachomatis infection in asymptomatic men. *Am. journal preventive medicine* **24**, 36–42 (2003).
2. EL Korenromp, et al., What proportion of episodes of gonorrhoea and chlamydia becomes symptomatic? *Int. journal STD & AIDS* **13**, 91–101 (2002).
3. E Williams, DA Williamson, JS Hocking, Frequent screening for asymptomatic chlamydia and gonorrhoea infections in men who have sex with men: time to re-evaluate? *The Lancet Infect. Dis.* **23**, e558–e566 (2023).
4. SM Jenness, et al., Incidence of gonorrhea and chlamydia following human immunodeficiency virus preexposure prophylaxis among men who have sex with men: a modeling study. *Clin. Infect. Dis.* **65**, 712–718 (2017).
5. G Rozhnova, et al., Elimination prospects of the dutch hiv epidemic among men who have sex with men in the era of preexposure prophylaxis. *Aids* **32**, 2615–2623 (2018).
6. G Rozhnova, MFS van der Loeff, JC Heijne, ME Kretzschmar, Impact of heterogeneity in sexual behavior on effectiveness in reducing hiv transmission with test-and-treat strategy. *PLoS computational biology* **12**, e1005012 (2016).
7. MA Kesler, et al., Actual sexual risk and perceived risk of hiv acquisition among hiv-negative men who have sex with men in toronto, canada. *BMC public health* **16**, 1–9 (2016).
